# PReS-FINAL-2140: Neutropenia with Tocilizumab (TCZ) treatment is not associated with increased infection risk in patients with systemic juvenile idiopathic arthritis (SJIA)

**DOI:** 10.1186/1546-0096-11-S2-P152

**Published:** 2013-12-05

**Authors:** F De Benedetti, HI Brunner, E Baildam, R Burgos-Vargas, G Horneff, HI Huppertz, K Minden, BL Myones, K Onel, J Wang, K Bharucha, D Lovell, A Martini, N Ruperto, PRCSG and PRINTO

**Affiliations:** 1 IRCCS Ospedale Ped Bambino Gesú, Rome, Italy; 2 PRCSG, Cincinnati, USA; 3 PRINTO, Genoa, Italy; 4 Roche, Welwyn, UK; 5 Genentech, S San Francisco, USA

## Introduction

In the phase 3 TENDER trial of TCZ in patients with sJIA, decreases in neutrophil count were commonly observed.

## Objectives

To determine if neutropenia was associated with increased risk of infection and to investigate variables associated with development of neutropenia in patients treated with TCZ for up to 2 years in TENDER.

## Methods

112 children with active, persistent sJIA were randomised 2:1 to receive TCZ by body weight (12 mg/kg <30kg or 8 mg/kg ≥30kg) or placebo IV every 2 weeks for 12 weeks and continued in an ongoing, TCZ open-label extension.[1]. Worst common toxicity criteria (CTC) neutropenia grade (grade 1, ≥1.5 and <2.0 × 10^9^/L; grade 2, ≥1.0 and <1.5 × 10^9^/L; grade 3, ≥0.5 and <1.0 × 10^9^/L; grade 4, <0.5 × 10^9^/L) and lowest observed neutrophil count (10^9^/L) were identified for each patient. Univariate linear regression analysis was performed to investigate association of patient characteristics with lowest observed neutrophil count. Rates of infections and serious infections (per 100 patient years [PY]) in periods ± 15 days around grade1-2 neutropenia (22.9 PY) and around grade 3-4 neutropenia (5.5 PY) were compared to corresponding rates in periods with normal neutrophil count (173.6 PY).

## Results

Up to week 104, 64/112 patients (57.1%) had at least 1 episode of grade 1-4 neutropenia; worst grade: 1 (n = 2), 2 (n = 34), 3 (n = 26) and 4 (n = 2). Rates of infections and serious infections during period of normal neutrophil counts (276.5/100PY [95% CI 252.3, 302.3] and 11.5/100PY [95% CI 7.0, 17.8], respectively) were comparable to those observed ±15 days around grade 1-2 neutropenia (226.7/100PY [95% CI 169.3, 297.3]; 8.7/100PY [95% CI 1.1, 31.5]) and grade 3-4 neutropenia (292.5/100PY [95% CI 167.2, 475.0]; 0/100PY), with no trend towards increased risk with higher grade neutropenia. Methotrexate (MTX) use (Yes/No) was significantly associated with lowest observed neutrophil count (difference: -0.575 [95% CI: -1.02, -0.13], *p *= 0.012), with 62% of 77 patients receiving MTX vs 46% of 35 patients not receiving MTX having grade 1-4 neutropenia. Younger age was borderline associated with lowest observed neutrophil count (β = 0.04661, *p *= 0.047). Concurrent use of glucocorticoids (GC) and TCZ exposure were not associated with lowest observed neutrophil count (*p*>0.3).

## Conclusion

No trend for association between neutropenia and increased risk of infections was observed in the TENDER trial. Background MTX, and somewhat younger age, was associated with increased risk for neutropenia, while TCZ exposure and concurrent GC use were not.

## Disclosure of interest

F. De Benedetti Grant / Research Support from: Abbott, Pfizer, BMS, Roche, Novimmune, Novartis, SOBI, H. I. Brunner Consultant for: Novartis, Genentech, MedImmune, EMD Serono, AMS, Pfizer, UCB, Janssen, Speakers Bureau: Genentech, E. Baildam: None Declared, R. Burgos-Vargas Grant / Research Support from: Abbott, Consultant for: Abbott, BMS, Janssen, Pfizer, Roche, Speakers Bureau: Abbott, BMS, Janssen, Pfizer, Roche, G. Horneff Grant / Research Support from: Abbott, Pfizer, H. I. Huppertz Consultant for: Abbott, Chugai, Pfizer, Roche, Swedish Orphan, K. Minden Grant / Research Support from: Pfizer, Abbvie, Consultant for: Pfizer, Abbvie, Roche, Chugai, Medac, B. L. Myones: None Declared, K. Onel: None Declared, J. Wang Employee of: Roche, K. Bharucha Employee of: Genentech, D. Lovell Grant / Research Support from: NIH, Consultant for: AstraZeneca, Centocor, Janssen, Wyeth, Amgen, Bristol-Meyers Squibb, Abbott, Pfizer, Regeneron, Hoffmann-La Roche, Novartis, Genentech, Speakers Bureau: Roche, Genentech, A. Martini Grant / Research Support from: Abbott, AstraZeneca, BMS, Centocor, Lilly, Francesco Angelini, GSK, Italfarmaco, MerckSerono, Novartis, Pfizer, Regeneron, Roche, Sanofi Aventis, Schwarz Biosciences, Xoma, Wyeth, Consultant for: Abbott, AstraZeneca, BMS, Centocor, Lilly, Francesco Angelini, GSK, Italfarmaco, MerckSerono, Novartis, Pfizer, Regeneron, Roche, Sanofi Aventis, Schwarz Biosciences, Xoma, Wyeth, Speakers Bureau: Abbott, Boehringer, BMS, Novartis, Astellas, Italfarmaco, MedImmune, Pfizer, Roche, N. Ruperto Grant / Research Support from: Abbott, AstraZeneca, BMS, Centocor, Lilly, Francesco Angelini, GSK, Italfarmaco, MerckSerono, Novartis, Pfizer, Regeneron, Roche, Sanofi Aventis, Schwarz Biosciences, Xoma, Wyeth, Consultant for: Abbott, AstraZeneca, BMS, Centocor, Lilly, Francesco Angelini, GSK, Italfarmaco, MerckSerono, Novartis, Pfizer, Regeneron, Roche, Sanofi Aventis, Schwarz Biosciences, Xoma, Wyeth, Speakers Bureau: Abbott, Boehringer, BMS, Novartis, Astellas, Italfarmaco, MedImmune, Pfizer, Roche.
